# Evaluation of a biplanar diode array dosimeter for quality assurance of step‐and‐shoot IMRT

**DOI:** 10.1120/jacmp.v10i4.3080

**Published:** 2009-09-30

**Authors:** Vladimir Feygelman, Kenneth Forster, Daniel Opp, Görgen Nilsson

**Affiliations:** ^1^ Division of Radiation Oncology H. Lee Moffitt Cancer Center Tampa Florida USA; ^2^ Department of Physics University of South Florida Tampa Florida USA; ^3^ ScandiDos AB Uppsala Sweden

**Keywords:** radiation therapy, intensity‐modulated radiation therapy, dose distribution comparison, three‐dimensional dosimetry, diode array dosimeter

## Abstract

In this paper, we describe and characterize a novel biplanar diode array, and demonstrate its applicability to dosimetric QA of step‐and‐shoot IMRT. It is the first commercially available device of its kind specifically designed for performing measurements at varying gantry angles. The detector consists of a cylindrical PMMA phantom with two orthogonal detector boards. There are a total of 1069 *p*‐type 1 mm wide diode detectors covering the measurement area of $20\times 20\;{\text{cm}}^{2}$ in each of the measurement planes. The orthogonal detector arrays ensure that the dose modulation information is not lost regardless of the beam incidence angle. For absolute calibration, the dose to the reference detector is calculated at the appropriate SSD and radiological depth by the treatment planning system and is scaled by the measured accelerator output. The directly measured rotational response on the central axis shows the maximum variation of approximately ± 3% in the narrow ±1º angular intervals centered on the detector boards. This variation is reduced to less than ± 2% outside of the four similarly centered ± 5% angular intervals. For all detectors, the difference between the measured and the calculated dose for a plan with 12 equally spaced beams is −0.2±0.9%. Of eleven IMRT plans, ten passed the γ (3%,3 mm) criterion at or above 95%, while one passed at 92%. The biplanar diode array is a useful tool for IMRT QA, allowing for essentially instantaneous online analysis of absolute dose errors in 3D.

PACS number: 87.55Qr

## I. INTRODUCTION

Early on in the development of clinical IMRT processes it was well understood that “at the heart of acceptance testing and commissioning procedures are dose measurements and their comparison with IMRT planning system calculations”.^(1)^ While historically this function was performed with an ion chamber for absolute dosimetry at a few points and with radiographic film for dose distribution verification, a number of alternative approaches emerged in recent years. A hypothetical ideal dosimeter for IMRT QA was described by Nelms et al.^(2)^ It should consist of very small (submillimeter), isotropic, absolute dose detectors arranged in a high‐density three‐dimensional array in a water‐equivalent phantom. The authors further point out that in order to be efficient and practical in a modern clinic, the device must provide online readout as opposed, for example, to film, which requires the additional postirradiation steps of development and scanning. Of course, all the traditional requirements for a good dosimeter apply as well – such as linearity, reproducibility, and energy independence.

That ideal IMRT dosimeter has not been built yet. The traditional ion chamber measurements can routinely yield a few measurement points at best. To obtain detailed spatial information, radiographic^(3)^ or radiochromic^(^ ^4^ ^–^ ^6^ ^)^ films have been employed, but both have limitations as a precise absolute dosimeter. While any film is essentially a two‐dimensional radiochemical dosimeter, it is understood that, ideally, three‐dimensional dose distributions require volumetric verification. To that end, a number of 3D radiochemical dosimeters with different formulations^(^ ^7^ ^–^ ^11^ ^)^ were introduced. The dosimetric advantages are potentially submillimeter resolution, water equivalency, and isotropic response.^(9)^ However, 3D radiochemical dosimeters require a rather complex postirradiation readout process, whether by an MRI or optical CT scanner. A number of two‐dimensional electronic detector arrays were introduced in recent years. Among the three popular commercially available devices, one is diode‐based^(^ ^12^ ^,^ ^13^ ^)^ and the other two utilize ionization chamber arrays.^(^ ^14^ ^,^ ^15^ ^)^ The common tradeoff with such devices compared to film is the ease of absolute two‐dimensional dose measurements at the expense of resolution loss due to both detector size and interdetector spacing. Poppe et al.^(16)^ analyzed the influence of those parameters on accuracy of IMRT dose measurements and concluded that dose variations in realistic IMRT dose distributions contain very little, if any, spatial frequency components above 0.1 mm^−1^. Therefore, the maximum detector spacing still compatible with accurate representation of the actual pattern of measured dose values in the sense of the Nyquist sampling theorem,^(^ ^17^ ^,^ ^18^ ^)^ was estimated at 5 mm (sampling frequency 0.2 mm^−1^). The three electronic arrays vary in detector size and spacing but share the same design approach of being 2D dosimeters originally intended for irradiation with the beam central axis perpendicular to the detector plane. However, the advent of dynamic rotational treatments with either conventional linacs or helical tomotherapy, resulted in emerging use of such devices for irradiation from multiple angles, as described in detail by Van Esch et al.^(19)^ and briefly by Woo.^(20)^ Among the challenges of such measurements are rotational dependence of the detector response and complex inhomogeneous structure of the entire device when viewed from the direction other than normal to the front surface. However, the most fundamental problem is that the amount of information about dose modulation across a beam is greatly reduced when the incidence direction changes from perpendicular to parallel to the array plane. Recently, a novel diode‐based detector (Delta4, ScandiDos AB, Uppsala, Sweden) was introduced and briefly characterized,^(^ ^21^ ^–^ ^23^ ^)^ indicating favorable day‐to‐day reproducibility, dose rate independence, and linearity. It is the first commercially available dosimeter array designed specifically for three‐dimensional dose verification of all currently available IMRT treatments, whether utilizing static gantry angles or dynamic rotational delivery. In this paper, we further examine the most clinically relevant properties of the Delta4 system, and describe the use of the detector for IMRT QA.

## II. MATERIALS AND METHODS

### A. Delta4 design

#### A.1 Hardware

**Figure 1:**
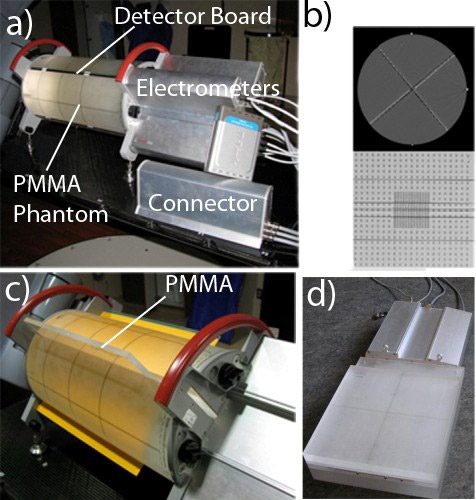
Delta4 dosimeter in the measurement position (a); CT scan of the device (b) with an axial slice through the center of the measurement region (top) and an oblique reconstruction through the midplane of the main detector board (bottom); the Delta4 phantom with film along the main board and the wings removed (c) with PMMA slab shown half‐inserted, for clarity; assembled calibration phantom (d) with the main detector board inserted.

The Delta4 device consists of a 22 cm diameter cylindrical polymethyl methacrylate (PMMA) phantom composed of separate pie‐shaped quarters, detector arrays with attached electrometers and data transmission electronics, and a connector unit (Fig. 1(a)). Each detector board is 10 mm thick, with the bulk of it (9.5 mm) being PMMA. In the center, there is a 0.5 mm thick fiberglass printed circuit board with the diodes soldered to the copper conductors. Portions of the PMMA are milled out to accommodate the diodes, which results in an array of air cavities visible on CT scans (Fig. 1(b)). The detector boards snugly fit between the phantom quarters during normal measurements. They can be temporarily removed from the phantom for calibration or repairs. The electrometers are connected to the portable network switch with Ethernet cables, and the switch is in turn directly connected to the dedicated network card in the control PC.

There are a total of 1069 *p*‐type cylindrical silicone diodes, whose active volume is 1 mm in diameter and 0.05 mm thick. The nominal detector sensitivity is 5 nC/Gy. Temperature sensitivity variation is reported by the manufacturer as 0.32%/°C, which compares favorably with the published temperature variation data for other *p*‐type diodes.^(24)^ The detectors are arranged in rectangular patterns (Fig. 1(b)) on two orthogonal planes. The first plane is called the “main board” and has the measurement area of $20\times 20\;{\text{cm}}^{2}$. The other plane is made up of two halves (“wings”), covering $20\times 10\;{\text{cm}}^{2}$ each, which allows for easy assembly of the detector boards in the orthogonal position in the phantom. The central line of detectors on the main board coincides with the long axis of the phantom. The detectors are spaced 0.5 cm apart in the central $6\times 6\;{\text{cm}}^{2}$ area, and 1 cm apart elsewhere. Rather than being angled $\pm 45^{\circ }$, the detector boards are separated from the vertical by +50° (main board) and −50° (wings) (Fig. 1(b)). The phantom can be positioned on the couch with the electrometers facing either away from the gantry (normal configuration depicted in Fig. 1(a)), or towards the gantry (reversed orientation). The operator must select the appropriate orientation in the software to ensure correct interpretation of the results. Since the bisector of the detector planes is offset from vertical, reversing the orientation of the phantom allows one to change the angle between the radiation beams and the detector boards by 10°. This provides additional flexibility in selecting the phantom orientation that avoids, as far as possible, beam incidence along a detector plane for nonrotational treatments. The system automatically suggests the best orientation based on the gantry angles in each plan.

For conventional linear accelerator measurements with fixed gantry angles, the control unit receives the trigger signal from the accelerator console. This trigger signal is available from the pulse repetition frequency coaxial test point connectors for the three major accelerator brands prevalent in the US. The device is designed to handle pulse repetition frequencies from 50 to 600 Hz. The trigger pulse precedes the dose pulse by a few microseconds. The detector samples the data only during a short time window just before, during, and after the dose pulse. Depending on the accelerator, integration starts 5–20 μs before the dose pulse and lasts for 60–100 μs. All channels are read out simultaneously and reset after each pulse. Readings over several dose pulses are packaged and sent to the control PC. This synchronization of the measurement with the accelerator pulse improves the signal to noise ratio, and adds the temporal dimension to the data, allowing, for example, the association of the dose‐packages with individual control points (segments) of the IMRT plan. For rotational treatments on conventional linacs, additional gantry angle information is obtained by Delta4 from an independent inclinometer mounted on the gantry.

#### A.2 Calculation model and software

Reference treatment planning data are transferred to Delta4 as DICOM RT objects, such as dose (calculated in the phantom), plan (beam arrangements), and structures from the original patient plan. Dose can be imported in RTOG format as well. The dose matrix, in principle, can be imported at the fraction, beam, and segment (for step‐and‐shoot IMRT) levels. While the DICOM receiver is designed to carefully check and automatically associate different objects to ensure that they belong to the same plan, a special mode allows the user to manually import the files and associate them with the selected existing objects in the system. This is necessary, for example, when the treatment planning system (TPS) is not capable of exporting the dose for each individual beam, and multiple plans with only one beam per plan need to be exported as a workaround.

In measurement mode, raw readings are converted to dose by applying a number of correction factors. Diode‐specific relative calibration factors obtained during the relative calibration routine are applied to account for the inherently varying sensitivity of the detectors. The optional temperature correction is applied equally to all diodes and accounts for possible temperature difference between the time of absolute calibration and measurement. The directional dependence corrections account for both the beam (gantry) angle and the diode's position along the phantom longitudinal axis. The directional correction factors were measured for 21 randomly selected diodes and averaged. Since the active volume is cylindrical, rotational dependence with respect to both symmetry axes was evaluated to allow for independent axial and radial corrections described above. Nillson^(21)^ reported the numerical range of the gantry angle rotational corrections to be 0.958–1.022 for a 6 MV beam. The depth and field size corrections were derived^(21)^ by comparison with the ion chamber measurements in a fat PMMA phantom. The field size correction varied from 0.978 to 1.009 in the range of field sizes from $5\times 5\;{\text{cm}}^{2}$ to $20\times 20\;{\text{cm}}^{2}$. The depth correction varied from 0.993 to 1.004 for the range of depth encountered in the Delta4 phantom (1.5–20.5 cm). The field size and depth corrections essentially compensate for the varying amount of low‐energy scattered photons in the beam. In summary, rotational, depth, and field‐size corrections are applied on a segment‐by‐segment basis to every individual diode in the direct beam, depending on the beam angle, the diode radial position, the segment equivalent field size, and the measurement depth. Correction factors are embedded in the software and are neither editable by, nor visible to, the end user under normal operating conditions. However, they can be read for each diode with the service software, and examples are presented in the Results section below.

The fundamental characteristics of the Delta4 diode detectors were studied previously. Nillson^(21)^ demonstrated that for the average of 100 detectors, the reading per monitor unit varied by no more than 0.1% between 10 and 100 MU. Dose per pulse dependency was studied by varying the SDD from 86.5 to 260.5 cm, resulting in the dose range from 0.18 to 0.02 mGy/pulse. The ratio of the diode reading to the ion chamber (corrected for recombination) varied by no more than 0.5%. In addition to confirming these results, Sadogapan et al.^(22)^ reported negligible dose rate dependency in terms of MU/min, and good short‐term and long‐term reproducibility. In particular, five four‐field box measurements over a three‐month period showed the standard deviation in reported dose of 0.6%. The user manual estimates long‐term sensitivity loss of the diodes at 1% per kGy of 6 MV radiation.

The system has a variety of tools for displaying differences between the measured and calculated (reference) dose. Specifically, for each measurement position *r*, the relative dose difference is defined as

(1) $$\Delta D_{\text{rel}}\;(\%)=100\%\times \frac{D_{m}(r)-D_{c}(r)}{D_{\text{nor}m}}$$

where indices *m* and *c* refer to the measured and calculated (planned) values, respectively, and $D_{\text{norm}}$ is the user‐selectable “normalization” dose value, having the same meaning as reported by Jursinic et al.^(12)^

The γ analysis is performed according to the formulae described in the original paper by Low et al.^(25)^ The acceptable dose‐difference $\left(\Delta D_{\text{Acc}}\right)$ has the same value for all evaluated points, as described originally.^(25)^ It is presented in the software as a user‐specified percentage of the “normalization” value $D_{\text{norm}}$, even though the Delta4 concept is built around absolute dose comparison. This leads to the apparent dependence of the γ analysis on the choice of the “normalization” dose, which has limited meaning beyond just being the arithmetical means to arrive at the (absolute) value of $\Delta D_{\text{Acc}}$. It can be different for the composite plan, individual beams, and segments.

If the treatment planning system only exports the composite dose matrix for a whole fraction, the system will display the common dose‐comparison analysis elements in two dimensions, for the two detector planes. If, in addition, the reference dose matrices for individual beams are present, the Delta4 has tools for a semiempirical volumetric dose calculation on both beam and fraction levels. For this calculation, the incidence rays are traced from the source through the phantom. Any ray will intercept at least one, and usually both detector planes, yielding measured dose point(s) along that ray. The system then essentially renormalizes the TPS‐calculated depth dose along the ray to fit the measurement point(s), and uses those data to reconstruct the dose. Once the process is complete for all the rays in all the beams, a three‐dimensional semiempirical dose matrix is available for comparison with the reference data. This dose matrix is presented with the same spatial resolution as the reference dose from the TPS. At this point, the dose agreement can be evaluated visually on multiple axial CT slices, or by using graphical dose‐volume histogram comparisons.

When the room lasers coincide with the central axis marks on the phantom, the center of the measurement area is at the treatment isocenter. The phantom can be shifted in the radial direction by up to ±9cm. This is useful when it is necessary to measure fields longer than 20 cm, when the target area on the plan is not at the isocenter, or when it is desirable to shift the detector array relative to the MLC pattern. The operator must physically shift the phantom prior to measurement, and input the magnitude and direction of the shift into the software prior to data analysis.

### B. Device calibration

#### B.1 General

The calibration procedures are intended to be performed by the end user and take about an hour to complete. The unit is equipped with a calibration PMMA phantom (Fig. 1(d)). The calibration frame sits on top of the backscatter slab. Either the main board or two wing boards can be secured in the frame at the same time. A system of interlocking steering pins and notches ensures that the detector boards can fit in the frame in only one orientation. A calibration frame lock and an additional buildup slab reside on top of the detector board being calibrated. The calibration SSD is 95 cm and the physical depth from the top of buildup to the midplane of the detector board is 4.28 cm (4.86 cm water‐equivalent depth^(26)^).

#### B.2 Relative calibration (equalization)

For relative calibration (equalization), a wide‐field algorithm is employed, with readings at seven different phantom positions taken in a $26\times 26\;{\text{cm}}^{2}$ radiation field. This procedure establishes the basic sensitivity value for each diode detector relative to the automatically selected reference detector. From the end user point of view, the main difference from that disclosed by Simon et al.^(27)^ is that it involves only translations, but not rotations, of the detector board between the measurement steps. When the detector is shifted such that the central axis of the beam is close to the phantom edge, an additional PMMA slab is placed next to the phantom to ensure full scatter conditions.

#### B.3 Absolute calibration

With the detector board in the calibration phantom centered in the beam, another exposure with a $10\times 10\;{\text{cm}}^{2}$ radiation field is performed to establish the reference detector signal. After that, the user must supply the numerical value of the absolute dose delivered to the reference detector. A standard calibration of the accelerator is performed. The dose at the reference detector location is calculated by the TPS in the measurement geometry (95 cm SSD, physical depth 4.3 cm, water‐equivalent depth 4.9 cm). This value is scaled by the accelerator output and supplied to the software as the measured dose at the reference detector.

### C. Evaluation procedures

#### C.1 General

A Varian Trilogy (Varian Medical Systems, Palo Alto, CA) with a 120‐leaf Millenium MLC was used for Delta4 calibration, open beam measurements, and step‐and‐shoot IMRT delivery. All treatment plans were generated using Pinnacle v. 8.0 (Philips Medical Systems, Fitchburg, WI) treatment planning system (TPS). A 3 mm dose calculation grid was used. The properties of the Delat4 were studied in detail with the 6 MV beam. Additional calibration and IMRT measurements were performed with the 15 MV beam. For analysis with the Delta4, all beams were delivered with their planned gantry angles, except when otherwise specified. Statistical analysis was performed with GraphPad Prism v. 5.1 software package (GraphPad Software, San Diego, CA).

#### C.2 Validation of array calibration

Because of the 3D nature of the array assembly, cylindrical shape of the phantom, and position‐specific corrections applied to the individual detectors based on the knowledge of that shape, the choice of calibration validation techniques must be somewhat different for Delta4 compared to the single‐plane arrays.^(13)^ Three methods were employed.

First, the relative calibration was studied with radiographic film in the Delta4 phantom. One set of detectors at a time was removed and replaced with PMMA slabs (Figure 1(c)). Ready‐pack EDR2 film (Eastman Kodak Co., Rochester, NY) was placed next to the remaining detector and the four quarters of the phantom were held together tightly by the eccentric screws. The beams were perpendicular to the detector board and passed through the film first. The phantom assembly was irradiated first by an open $20\times 20\;{\text{cm}}^{2}$ beam and then with the 30° enhanced dynamic wedge (EDW), introducing dose gradient and dose rate variation across the field. Finally, a composite treatment consisting of seven segmented IMRT beams from a head‐and‐neck plan was delivered with the constant gantry angle. The films were scanned at a 356 μm per pixel resolution with a 16‐bit Vidar Dosimetry Pro digitizer (Vidar Systems Corp., Herndon, VA) driven by RIT software (RIT113 v. 5.1, Radiological Imaging Technology, Inc., Colorado Springs, CO). The film was calibrated in a standard manner.^(3)^ Absolute dose values were exported from Delta4 for either detector array in the form of a 16‐bit monochrome image file (TIFF) with 1 mm resolution. Along with this export, Delta4 generates a text file that defines a linear equation converting pixel values to dose. This equation was applied on a pixel‐to‐pixel basis utilizing an image manipulation routine in the ImageJ software package (ImageJ v. 1.41f, available in public domain from http://rsb.info.nih.gov/ij/). The resulting dose maps were compared to the film dose distributions in the RIT software.

The second method of validating the relative calibration was a modified 180° rotation test^(13)^ performed with the fully assembled phantom. The phantom was irradiated with a $25\times 25\;{\text{cm}}^{2}$ field once in the default position (electrometers away from the gantry – Fig. 1(a)) and then in the reversed orientation (electrometers toward the gantry). Because the bisector of the detector planes is angled 5° from the vertical, the gantry angle was set to 5° for the first measurement and 355° for the second one. This way the detector boards were symmetrical with respect to the beam in both cases. The data from the first measurement, appropriately transformed spatially, was used as the reference data for the second irradiation. The relative dose‐difference was computed for each diode

Finally, for the absolute calibration verification, the dose registered by the reference Delta4 diode in a single $18\times 18\;{\text{cm}}^{2}$ beam with the gantry angle of 5° was compared to the calibrated Isorad‐3 diode detector (Sun Nuclear Corp., Melbourne, FL). The Isorad detector was positioned at the center of the cylindrical phantom between the PMMA slabs replacing the detector boards. The calibration factor for the diode was obtained under similar irradiation conditions ($18\times 18\;{\text{cm}}^{2}$ field size and 10 cm depth) in Plastic Water (CIRS Inc., Norfolk, VA). It was noted that the Isorad diode exhibited higher readings in PMMA compared to the water‐equivalent plastic when placed at the same source to detector distance and at the appropriately scaled depth. This is due to excess scatter in acrylic,^(26)^ and a PMMA to water correction factor was established for the diode by direct intercomparison of the readings.

#### C.3 CT representation of the phantom for dose calculation

Two possible alternatives were evaluated for representing the Delta4 phantom in the treatment planning system. The first one was a synthetic homogeneous dataset. If this option is used, the user has to ensure that the synthetic CT number translates to the correct relative density. Although Pinnacle uses physical density, we employed the density of 1.14, corresponding to the electron density of PMMA relative to water.^(26)^ The second option was a physical CT image data set, which was obtained on a Philips Brilliance scanner (Philips Medical Systems, Cleveland, OH) in a helical mode with the reconstructed slice thickness of 1 mm and an axial resolution of 0.48 mm/pixel. The clinical CT to ED file was used with this dataset. Pixel statistics for the physical CT scan were evaluated with the ImageJ software.

#### C.4 Rotational dependence

The detectors on the central axis of the cylindrical phantom are most suitable for the rigorous evaluation of the detector sensitivity variation with gantry rotation. Two measurements were performed. First, the main board was placed in its normal position in the cylindrical phantom but the wing boards were replaced by two PMMA slabs. The phantom was irradiated at 15° gantry angle increments from 0 to 360°. The absolute dose readings of the three central diodes were averaged and corrected by the rotational dependence of the accelerator output measured with the Farmer chamber and a buildup cap. The data were normalized to the value with the gantry at zero degrees.

Second, the rotational dependence for the fully assembled phantom was measured. The typical gantry angle increment was 15° but the steps shrunk progressively to 1° as the beam incidence approached the direction along any detector board. The data were corrected for the rotational variation in accelerator output and normalized to the value corresponding to the gantry angle of 0°. Recorded dose values were plotted both as raw data (equivalent to the comparison with the homogeneous phantom calculations), and also as normalized to the expected values calculated on the CT data set. The mean dose to a small cylindrical volume around the three central diodes represented the calculated dose. The size of the target was roughly representative of the Farmer chamber collection volume.

For the detectors situated away from the phantom long axis, it is not easy to design a rigorous yet practical experiment to test the rotational dependence because the symmetry with gantry rotation is lost. However, it is reasonable to assume that because all the detectors are of the same design and are mounted in the same way, the rotational dependence would be similar. If this is the case, one should expect a good agreement between the measured and calculated dose for a series of beams approximating a 360° arc. We used 12 equally spaced $22\times 25\;{\text{cm}}^{2}$ beams. The field size was chosen to avoid sharp dose gradients in the measurement area, so that every point could be evaluated for dose‐difference. No beam was incident within ±5° angular intervals around any detector board.

#### C.5 Point dose verification of semi‐empirical calculations

A single beam measurement was performed for an $18\times 18\;{\text{cm}}^{2}$ beam at 5° gantry angle (equal 45° angles to both detector planes). Point doses were calculated on the central slice at different depths, along the fan rays passing directly through the central diode on the main board, two diodes on different detector boards, or one diode on either board. This was done to assess the accuracy of the three‐dimensional semiempirical dose determination with the different number of actual measurement points intercepted by a given ray. The calculated and measured doses were compared on the synthetic and CT phantom data sets. The dose agreement was also evaluated for an $18\times 18\;{\text{cm}}^{2}$ four‐field box at the cardinal gantry angles, and $18\times 18\;{\text{cm}}^{2}$ and $4\times 4\;{\text{cm}}^{2}$ beam arrangements at seven equally spaced gantry angles.

#### C.6 IMRT measurements

The commissioning results for the treatment planning system indicated 2%/2mm agreement for all the scanned data for the open fields. The calculated (absolute) dose profile for an MLC bar pattern^(28)^ did not deviate by more than 1% from the diode scan. The point doses measured with a microchamber, including the heavily blocked fields crucial to the IMRT dosimetry with a tertiary MLC, did not deviate from the calculations by more than 2%, with most points being within 1%.

One phantom‐based and six clinically representative IMRT QA plans were calculated on the Delta4 phantom with the 6 MV beam. Some of these cases were recalculated with the 15 MV beam. While not necessarily clinically relevant, this arrangement allowed for direct comparison of IMRT dosimetry for the two available X‐ray energies. Four clinical plans represented head and neck treatments, one prostate and one abdomen (Table 1. Three plans required at least some of the beams to be split into two because of the carriage shift due to the MLC leaf extension restrictions. The accelerator output was determined with an ion chamber in a Plastic Water phantom immediately prior to Delta4 measurements, and the Delat4 dose was scaled accordingly. All IMRT plans were also recalculated on a $20\times 20\times 20\;{\text{cm}}^{3}$ Plastic Water Cube phantom (CIRS) and point doses were measured with a 0.03 cm^3^ microchamber (PTW PinPoint 31015).

**Table 1:** Step‐and‐shoot IMRT: ion chamber dose error and y analysis for all detectors receiving ≥30% of the “normalization” dose.

*Plan*	*Plan Type*	*6 MV*		*15 MV*	
		*IC Dose‐Difference, %*	*γ(3%, 3mm)*	*IC Dose‐Difference, %*	*γ(3%, 3mm)*
1	Phantom (Prostate)	−0.2	100	–	–
2	Prostate	0.9	99.8	1.2	100
3	H&N	−0.9	100	1.2	99.8
4	H&N	−0.8	100	−0.2	100
5	H&N (split beam)	0.2	92.0	0.8	95.4
6	H&N (split beam)	−0.6	99.6	0.8	–
7	Abdomen (split beam)	0.5	95	1.5	–

## III. RESULTS & DISCUSSION

### A. Validation of array calibration

**Figure 2:**
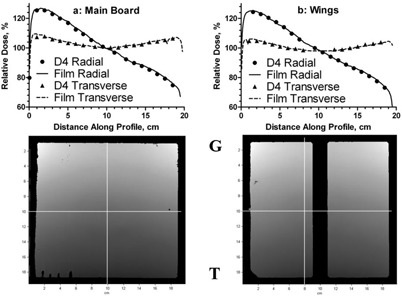
Orthogonal relative dose profiles for EDW fields obtained with film and Delta4 for the main detector (a) and wings (b). Dose maps below corresponding graphs show the areas where dose‐difference exceeds 2% (mostly dark “frames” on the periphery), and indicate the location of the profiles. “G” and “T” mark the gantry‐target direction.

Dose‐distribution analysis presented in Fig. 2 was performed in relative mode, with the dose distributions normalized at the respective crosshairs’ intersection points. The results are presented for the 30° EDW fields but are similar for the open beams. The dose‐difference threshold tool was chosen to present the data. The black areas on the dose maps represent the areas where film disagrees with the diode arrays by more than 2%. Disagreement is essentially confined to the penumbra region, where it is expected. The area of disagreement in the middle of the wings is an interpolation artifact resulting from the absence of the detectors there. It is present only when the dose image is exported for test purposes, but not during the normal use of the device. The “fingers” in the lower left corner of the main board dose map are due to a visible processing artifact. Of those pixels satisfying the 2% dose‐difference criterion, 84 and 92% for the main board and the wings, respectively, also pass at the 1% level. Orthogonal profiles extracted along the respective crosshairs also indicate good agreement between film and Delta4. It is estimated that EDR film agrees with the ion chamber to within 1% for relative measurements in the low gradient area of open fields^(29)^ or 2% for the IMRT fields.^(30)^

**Figure 3:**
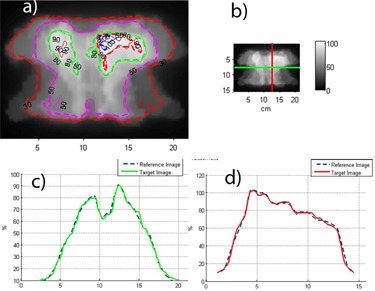
Relative dose comparison between film and Delta4: (a) Isodose overlay; (b) profile directions; (c) transverse profile ‐ Delat4 vs. film (green); (d) longitudinal profile (film is red).

Dose distributions for seven modulated beams delivered at the same gantry angle are compared between film and Delta4 in Fig. 3. This beam configuration was chosen because film cannot be placed closer than 5 mm to the center of the detector board, where the measurement points are located. A five millimeter distance can be significant in terms of the dose‐difference with IMRT. With all the beams perpendicular to the detector board and adjacent film, this error is minimized. Isodose distributions and dose profiles agree between film and the Delta4 for the segmented beams as well.

The mean dose difference for all detectors between the measurements in the default and reversed (rotated 180°) phantom orientations was0.06±0.6% (1SD). Ninety‐seven percent of the diodes were within 1%, with the maximum deviation of 1.4% (2 diodes). This experiment further validates relative calibration of the diodes on both detector boards by exchanging their position in the beam.

For the absolute dose in the center of the phantom, the monitor units were calculated to deliver 200 cGy. The central (reference) Delta4 diode registered 200.2 cGy. The Isorad diode reading, corrected for the overresponse in PMMA (0.981), was 198.8 cGy (0.7% lower). The Delta4 dose was close to the expected, with the independent diode dosimeter differing by less than 1%.

No formal study of the long‐term dose measurement stability was designed for this work as this has been done previously.^(22)^ However, we have a history of seven absolute calibrations of the device in the same beam over 12 months. The standard deviation of the calibration factor deviation from the mean is 0.9%.

### B. Rotational dependence

It is expected that the detector readings would exhibit directional dependence. This is due to the different effective depth to the active volume when the diode is irradiated from the opposite directions, which includes at a minimum the difference attributable to the detector board design (Fig. 1(b)).

**Figure 4:**
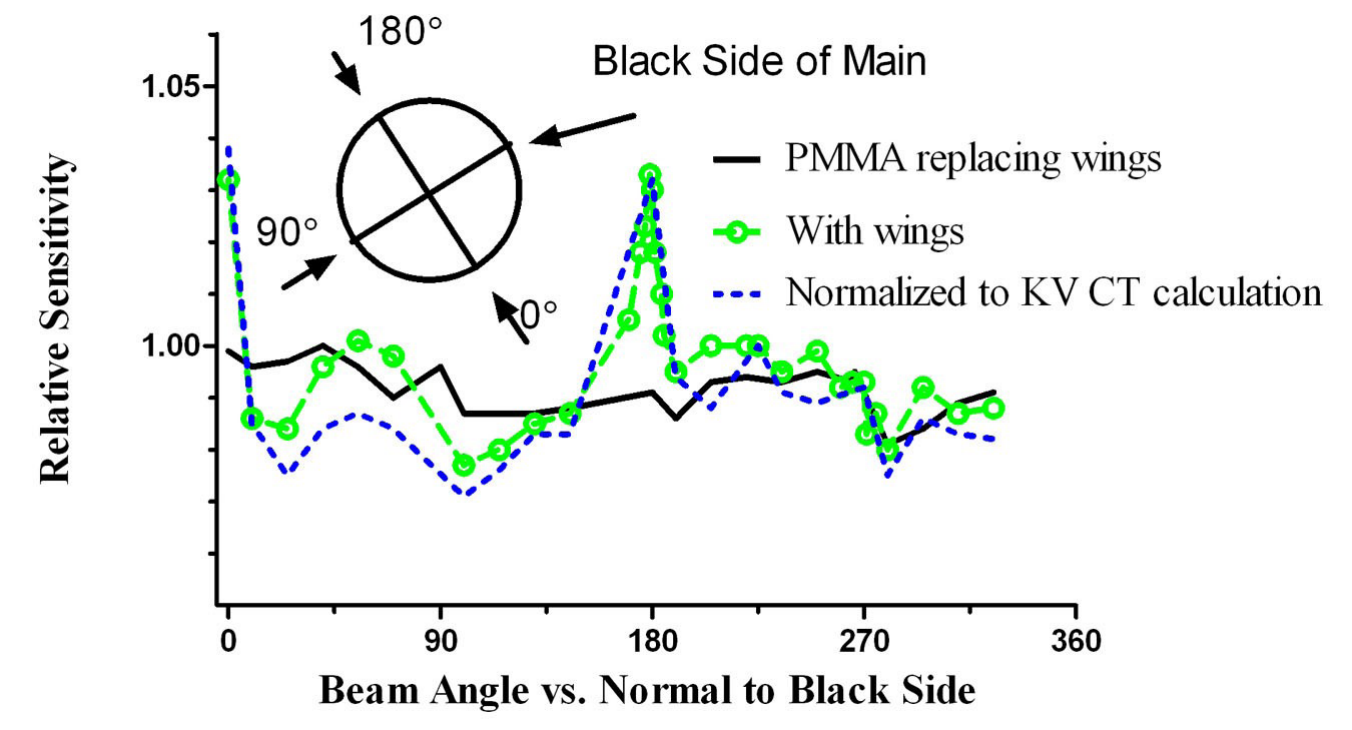
Relative sensitivity variation vs, the angle to the normal to the black side of the main board. Raw readings with PMMA replacing the wings, with wings in place, and readings normalized to the TPS‐predicted values on the CT dataset.

The graph of the average readings of the three central diodes with the wings replaced by PMMA is presented in Fig. 4. In such an arrangement, the data are most representative of the corrected directional sensitivity dependence of the diodes. Relative signal is expressed as a function of angle between the beam central axis and the normal to the black side of the main detector board. Normalized to the gantry angle at zero, the values vary from 0.981 to 1.000, with the mean of 0.991. The lowest value is associated with the beam central axis ten degrees away from being parallel to the detector board.

The rotational dependence data for a fully assembled device were analyzed in two different ways. In Fig. 4, the graphs are presented for the raw data (equivalent to comparison with the homogeneous phantom calculations), and corrected with the CT dataset. The mean values are 0.999 (range 0.977–1.033) without the correction, and 0.989 (0.971–1.038) for the CT dataset, respectively.

**Figure 5:**
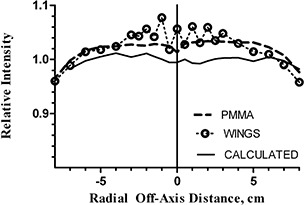
Radial beam profiles for a single beam: measured with PMMA replacing the wings, measured with the wings in place, and calculated by the TPS. Beam incidence is perpendicular to the main board (along the wings, when applicable). All profiles are normalized to the calculated value at the central axis.

While the rotational dependence experiments utilized the average dose from the three central diodes, we also examined a related issue of the shape of the radial beam profile with the beam passing directly along the wing detector board (Fig. 5) The relatively large periodic variations in point doses correlate qualitatively with the periodic variation in the detector board CT numbers (Fig. 1(b)). The effect is more pronounced in the central ±3cm area of the profile, corresponding to the region of the higher detector density. The calculation on the CT data set failed to predict this dose fluctuation. While apparent under test conditions deliberately designed to evaluate the limits of the device performance, this periodic dose error is not of great importance in clinical practice. To illustrate this point, we recorded the relative dose errors for the central 168 detectors on the main board, first with the beam parallel to the board, and then angled by 5°. The “normalization” point was at the isocenter. The mean relative error, standard deviation, and 95% range were 0.4±3.6(−6.7 to 6.4), and 0.0±1.2(−2.8 to 1.8)%, respectively. Let us examine a hypothetical case of a rotational treatment delivered by a uniform full arc. Let us further assume, conservatively, that the dose errors would add in the same direction, resulting in a 5% average error for any given diode when the beam is incident within the four ±5° angular beam intervals centered around the boards. Weighted by a factor of 40º/360º=0.11, it would lead to a cumulative 0.5% error attributable to the dose perturbations by the detector arrays. For fixed gantry angle IMRT, the design of the device permits avoidance of beam incidence along the detector boards altogether.

Projecting 12 equally spaced open beams on the cylindrical phantom resulted in the mean difference for all detectors between the measured and calculated dose of −0.21±0.91%(1SD).

Ninety‐seven‐percent of the measurement points were within 2%, and essentially all were within 3% of the predicted dose. While not as rigorous as the direct measurements on the phantom axis, these results support the assumption that the detectors away from the axis exhibit a similar rotational response.

### C. Point dose verification of semiempirical calculations and CT representation of the phantom

**Figure 6:**
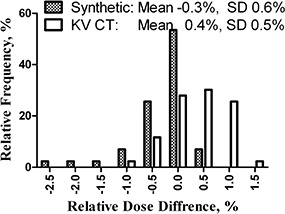
Histogram of relative measured vs. calculated (on synthetic and physical CT datasets) point dose differences for a single beam at 45° incidence angle to both detector planes.

The first open beam case utilized a single $18\times 18\;{\text{cm}}^{2}$ beam incident at 45° to both detector planes. The histogram of relative dose differences for a number of points along the different fan rays is presented in Fig. 6. The dose was calculated for both synthetic and CT datasets. The statistics for both datasets show similar standard deviations. The mean values deviate from zero by about the same amount in the opposite directions for the homogeneous synthetic phantom and the CT datasets. The difference between the means is statistically significant (t‐test, p<0.0001). For TPS calculations on the synthetic dataset, the bulk relative density was set to a nominal PMMA electron concentration value of 1.14. With the CT to ED tables used, the mean pixel values for the homogeneous portion of the Delta4 phantom corresponded to the relative density of 1.16±0.17. This difference in average densities correlates with the mean dose deviation magnitude and sign in Fig. 6.

The beam central axis effectively intercepted only one detector plane, while other fan rays intercepted both. Also, some evaluation points coincided with individual diodes. There was no statistical evidence that the mean dose deviation was different between the points on the central ray and the rest of the data (t‐test). The same holds true for the dose points coinciding with individual detectors compared to those where the measured dose was reconstructed (as described in Section II. A. 2. (t‐test)).

Calculated doses for the subset of points coinciding with individual detectors deviated from the measurement by −0.8 to0.6%. The range of correction factors (excluding temperature) applied to those detectors was from 0.960 to 1.011. This spread of correction factors is primarily due to the variation in rotational dependence (0.991– 1.039). The range brackets the 1.0 value because the wings are oriented towards the beam opposite each other – when the diode side faces the beam on one wing, the PCB side faces the other. Without the corrections, dose‐error would have ranged from −4.7 to0.8%. As an example, the individual component factors for the central diode are 1.023 for rotational dependence, 0.993 for depth, and 0.972 for field size.

The single beam and four multiple‐beam arrangements were analyzed by common dose‐comparison metrics of dose difference, distance to agreement, and γ analysis,^(25)^ with the formulae presented in Methods above (Table 2. The single beam dose was normalized to the maximum value, while the multiple‐beam plans were normalized to isocenter. Detectors that received at least 30% of the normalization dose were included in the analysis. The subset of those in the region with dose gradient of at least 3%/mm was included in the DTA analysis. The superior agreement for a single beam compared to those of the four‐field box and both seven beam arrangements was largely due to the difference in “normalization” levels. As described in Methods, the choice of “normalization” value affects the results of Γ analysis. While the choice of a single $\Delta D_{\text{Acc}}$ is relatively straightforward for an isocentric set of beams or even for a single beam perpendicular to the dosimeter array, it is non‐trivial for the beams with a substantial incidence component along the detector board. In the latter case, a substantial number of points in the relatively low gradient area will be analyzed using an either overly permissive or too stringent dose‐difference criterion, depending on the location of the “normalization” point on the PDD.

The measurements for seven‐beam plans were made under conditions that could potentially be experienced in routine clinical use. To explore the worst case scenario, in both cases one beam entered along the detector board, producing periodic discrepancy in the radial profile similar to that in Fig. 5. Inspection of the dose‐error spatial distribution confirms that the dose‐errors in excess of 3% are largely confined to the periphery of the irradiated area, where distance‐to‐agreement is a more appropriate criterion. This results, for example, in the 94.8% γ(3%,2 mm) passing rate for the seven $18\times 18\;{\text{cm}}^{2}$ beams. Under optimal conditions (the 12 beam plan in Table 2), with no beams incident along the detector boards and low dose gradient, 99.8 of the diodes read within 3% of the expected value.

Although the phantom contains some high atomic number materials, their volume is sufficiently small to produce minimal CT artifacts (Fig. 1(b)). The maximum pixel value in the dataset is 288, which is well within the range of the practical CT to ED conversion table. From the data presented in Fig. 6 and Table 2, the calculations performed using the actual CT dataset show that it can be used as well.

**Table 2:** Dose‐difference analysis (dose, distance to agreement and γ) for open beam plans.

*Beam Arrangement*	*Synthetic, % pass* ^a^			*CT, % pass* ^a^, ^a^		
	*Dose (3%)*	*DTA (2 mm)*	*γ(3%, 2 mm)*	*Dose (3%)*	*DTA (2 mm)*	*γ(3%,2 mm)*
$5{}^{\circ},18\times 18\;{\text{cm}}^{2}$	99.2	75.0	99.3	99.3	80.6	99.7
Four‐field box, $18\times 18\;{\text{cm}}^{2}$	87.6	100	94.3	91.7	100	97.1
Seven equally spaced fields, $18\times 18\;{\text{cm}}^{2}$	72.0	100	87.9	85.0	100	94.8
Seven‐field, $4\times 4\;{\text{cm}}^{2}$	64.7	92.9	92.2	71.6	92.1	94.0
Twelve $25\times 25\;{\text{cm}}^{2}$ beams	99.8	N/A	99.8	N/A	N/A	N/A

^a^ Dose difference is reported for all detectors receiving more than 30% of the “normalization” dose. DTA is reported for the subset of detectors in the region with dose gradient of at least 3%/mm.

### D. IMRT measurements

The choice of “normalization” dose affects the results of γ analysis as implemented in the Delta4 software (Low et al.^(25)^). To have a meaningful comparison between different plans, it is important to establish a logical and consistent way of defining the “normalization” dose. The dose to isocenter is not sufficiently predictable for IMRT because of dose inhomogeneity. The choice of prescription (PTV) dose would lead to results variability, depending on the patient size compared to the phantom. We decided to take advantage of the device's DVH display capability and defined the normalization dose as the minimal dose to 95% of the PTV volume, as calculated by the TPS on the Delta4 phantom.

The relative dose difference statistics and the corresponding γ analysis results for 11 IMRT plans calculated on the homogeneous dataset are presented in Table 1. The low‐dose cutoff for analysis was set at 30% of the “normalization” value. It was experimentally verified that this leads to more stringent gamma analysis than setting the cutoff dose at, say, 10%. With the global dose threshold, low‐dose points tend to automatically pass on dose, thus inflating the passing rate. We report the results for the 3%/3mm parameter combinations, which appear to become a *de facto* standard^(31)^ for the electronic arrays. The gamma analysis results are quite satisfactory, considering that the Delta4 accuracy (estimated in the user manual at 2%) and the TPS accuracy (2%) add in quadrature to 2.8%.

## IV. CONCLUSIONS

The Delta4 device is capable of quickly providing a large amount of absolute, three‐dimensional dose data, which makes it a robust tool for QA of IMRT dosimetry. As with any complex tool, its capabilities and limitations must be evaluated in relation to the specific clinical situation. While absolute doses are compared, the choice of dose “normalization” (in our opinion, better termed “reference”) level affects the numerical results of γ analysis. To ensure consistency in comparing the results for different size patients, we chose the minimum dose to 95% of the PTV, as calculated on the phantom, to be the standard “normalization” value. Good understanding of the delivery mechanics would help to optimize data collection and analysis. This paper was limited to evaluating the basic features of the device and to static IMRT delivery modes. Other options, such as DVH analysis, segment‐level information, or applications to dynamic rotational treatment delivery will be investigated in the future.

## ACKNOWLEDGEMENTS

The authors wish to thank Dr. Dung‐Tsa Chen for his advice on statistical analysis, and Dr. Geoffrey Zhang for helpful discussions. One of the authors (G.N.) is the president and CEO of ScandiDos, which provided the Delta4 unit for evaluation at no charge.
